# Promiscuity and specificity in ligand sensing by plant cell surface receptors

**DOI:** 10.1371/journal.ppat.1013548

**Published:** 2025-10-08

**Authors:** Christina E. Steidele, Julien Gronnier, Martin Stegmann, Ralph Hückelhoven, Martina K. Ried-Lasi

**Affiliations:** 1 Chair of Phytopathology, TUM School of Life Sciences, Technical University of Munich, Freising, Germany; 2 Chair of Plant Cell Biology, TUM School of Life Sciences, Technical University of Munich, Freising, Germany; 3 Institute of Botany, Molecular Botany, Ulm University, Ulm, Germany; 4 Department of Molecular Signal Processing, Symbiosis Signalling, Leibniz Institute of Plant Biochemistry, Halle (Saale), Germany; University of Tübingen: Eberhard Karls Universitat Tubingen, GERMANY

## Introduction

Plants rely on cell surface receptors to detect self, non-self, and modified-self molecular patterns, shaping their responses to microbial encounters. Drawing on recent selected case studies, we explore the complexity of pattern recognition. Despite structural similarities, many receptors have evolved striking specificity, distinguishing subtle variations in ligand sequences and modifications. Across this diversity, common themes emerge: convergent receptor evolution, functional integration of growth and immunity, dual roles of peptides as both structural and signaling elements, receptor versatility in recognizing diverse peptide ligands, and context-dependent signaling mediated by shared receptor complexes. This review highlights recent advances and open questions on how receptor and ligand diversity contribute to output specificity in plant signaling.

## RALF peptides—Dual-function peptides bridging structure and signaling

**Fig 1:**
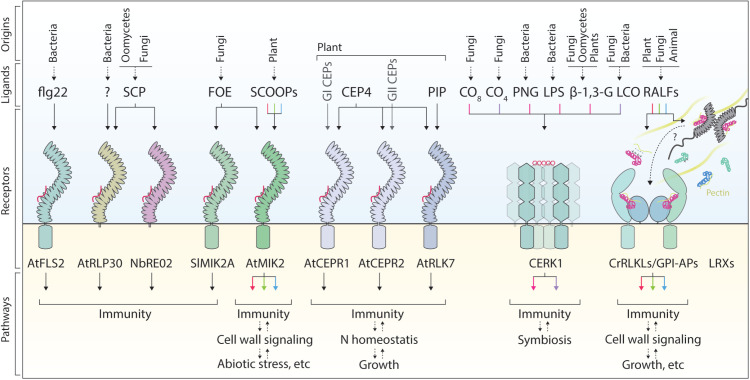
Promiscuity and specificity in ligand recognition by immunoregulatory cell surface receptors. Plant PRRs vary widely in their ligand recognition spectrum. Some, like Arabidopsis FLS2, exhibit high specificity by recognizing a specific allele of the bacterial flagellin epitope flg22. Others, such as AtMIK2, are more promiscuous, perceiving a vast array of related or unrelated ligands, including members of the large Brassicales-specific SCOOP peptide family and the *Fusarium oxysporum* elicitor (FOE), thereby regulating diverse physiological processes. CrRLK1L/GPI-AP complexes (e.g., FER–LLG1) recognize both endogenous and pathogen-derived RALF peptides. Convergent evolution has led to unrelated receptors such as AtRLP30 and *Nicotiana benthamiana* RE02 independently perceiving SCP from fungi and oomycetes. RALF peptides also serve as ligands for multiple receptor complexes, including LRXs and CrRLKLs-GPI-APs. Notably, AtRLP30 also detects distinct bacterial patterns, enabling recognition across fungal, oomycete, and bacterial kingdoms. Phylogenetically related receptors (e.g., CEPR1, CEPR2, RLK7) show subclade-specific ligand preferences, with group I CEPs perceived primarily by CEPR1, while peptides like CEP4 are redundantly recognized by CEPR1/2 and RLK7. Co-receptors such as CERK1 confer broad versatility and are engaged for the perception of a plethora of molecules, with CERK1 involved in perceiving diverse plant and microbial saccharides, coordinating both immune and symbiotic signaling. The colored lines, arrows, and RALF peptides represent the specific relationships between ligands and their signaling outputs. Red, immunity; green, cell wall signaling; blue, abiotic stress/growth; lilac, symbiosis. Abbreviations: CEP, C-terminally encoded peptide; CERK1, chitin elicitor receptor kinase 1; LRX, leucine-rich repeat extensin; RALF, rapid alkalinization factor; SCP, small cysteine-rich protein.

Paradigmatically, secreted peptides function as ligands for specific classes of cell surface receptors, initiating defined signaling pathways. Remarkably, the Rapid alkalinization factors (RALFs), a class of cysteine-rich plant peptides, associate with several unrelated receptors, serve both structural and signaling functions, and play pivotal roles in immunity, growth, and reproduction [1–3]. Common principles underlying RALFs modes of action have recently emerged. As illustrated by RALF1, RALF4, RALF22, and RALF23, RALFs possess a net positive charge and bind pectic homogalacturonan, a major cell wall component [4–8]. RALF-pectin oligomers associate with leucine-rich repeat extensins (LRXs) proteins at the cell wall, contributing as structural elements to cell wall integrity (Fig 1) [4,5,9].

At the plasma membrane, RALFs nucleate the assembly of heterotypic receptor complexes that include the glycosylphosphatidylinositol-anchored proteins (GPI-APs), LORELEI and LORELEI-like GPI-APs, together with *Catharanthus roseus* receptor-like kinase1-like (CrRLK1L) family members (Fig 1) [10], initiating signaling events that often result in rapid apoplastic alkalinization [11–13]. Pectin plays a central role in RALF perception at the plasma membrane [6–8], promoting the formation of RALF-pectin molecular condensates proposed to modulate the nano-organization of membrane proteins [6,14,15] and condition RALF-induced signaling [7,8]. Notably, single RALFs can bind pectin, cell wall, and plasma membrane-located receptors [5,14,16,17]. RALF interactions with LRXs and GPI-APs involve distinct and mutually exclusive binding modes [9,10], and a flexible region within GPI-APs determines RALF binding specificity [10].

RALF perception can involve multiple CrRLK1Ls and GPI-APs, as shown in reproduction, where four CrRLK1Ls participate in RALF22 perception [17,18]. Whether structural determinants that define RALF-CrRLK1L binding specificity exist remains unknown. Likewise, the structural basis for the formation of higher-order CrRLK1Ls/GPI-APs complexes is unclear. Additionally, RALFs can act antagonistically, competing for the same receptor, and a single RALF can elicit opposite effects in a concentration-dependent manner [17,19,20]. The surface charge varies among RALFs and may specify affinity for pectin methylation patterns, a potential “RALF-pectin code” which could fine-tune RALFs structural and signaling functions. Further, depending on their identity RALFs could recruit specific receptor complex components and signaling proteins in a charge, pectin methylation status, and/or concentration-dependent manner to induce distinct downstream response outputs.

## MIK2—Single-receptor versatility for diverse peptide ligands

While many small secreted peptides signal through related RLKs, exceptions exist where a single receptor mediates all responses to one class of peptides. The Male discoverer 1-interacting receptor-like kinase 2–Serine-rich endogenous peptide (MIK2–SCOOP) module exemplifies this: SCOOPs are Brassicales-specific phytocytokines with over 50 peptides predicted in Arabidopsis [21–24]. The LRR-RLK MIK2 is the sole SCOOP receptor (Fig 1), as *mik2* mutants are insensitive to SCOOPs [21,22]. The MIK2–SCOOP module acts in plant immune responses, biotic and abiotic stress resistance, cell wall integrity sensing, senescence, and flowering time regulation [21,22,25–30]. Notably, MIK2 also mediates Arabidopsis responses to an unidentified peptide elicitor from **F*usarium *oxysporum, FOE,** and related fungi [26,31].

The ability of a single receptor to generate diverse signaling outputs may arise from the diversity of SCOOP ligands. A high number of SCOOPs with varying expression and affinities might have evolved because of a restricted number of corresponding receptors. *Vice versa*, the ability of SCOOPs to trigger strong seedling growth inhibition at low concentrations [21,22] might have restricted MIK2 gene duplications and diversifications in Brassicales. While MIK2 is ubiquitously expressed, its transcript levels are modulated by environmental cues. SCOOPs also show distinct expression patterns, suggesting that spatiotemporal co-expression of MIK2 with specific SCOOPs could confer signaling specificity [24,28,29]. Not all SCOOPs equally promote MIK2–co-receptor complex formation [21], and both individual and post-translationally modified SCOOPs exhibit distinct, sometimes antagonistic functions. The loss of single *PROSCOOP* genes can thus result in unique phenotypes that are not compensated by other family members, pointing to functional diversification [24,27–30]. It is not understood whether SCOOPs or SCOOP variants compete for MIK2 receptor binding. However, such a competition would enable fine-tuned combinatorial signaling “cocktails,” in which, rather a milieu of receptor ligands than a single ligand together with the abundance of the receptor and possible co-receptors at a single cell level determine thresholds for receptor activation or attenuation. Recent structural insights into the MIK2–SCOOP interface will help clarify functions of individual SCOOPs that share a central SxS motif but can otherwise be very diverse [23,32,33]. On top of this, MIK2 may be part of higher-order receptor complexes that may function independent of SCOOPs but generate signals that converge or interfere with SCOOP signaling, thereby creating context-dependent SCOOP signaling results.

Phylogenetic analyses reveal that SCOOPs and highly similar homologs of Arabidopsis MIK2 are restricted to Brassicales [21–23]. However, less similar but orthologous MIK2-clade RLKs occur in other plant families, which do not respond to SCOOPs. For instance, tomato MIK2A is a functional RLK seemingly responding to the same fungal elicitor as Arabidopsis MIK2 (Fig 1) [31]. Expansion of the MIK2 family in species lacking SCOOPs [31] raises questions about potential alternative exogenous or endogenous ligands and a ligand-receptor co-evolution beyond Brassicales.

MIK2 demonstrates how one receptor can mediate responses to a wide array of endogenous and microbial peptides, enabling broad-spectrum signaling specificity.

## SCP receptors—Convergent receptor evolution for conserved microbial patterns

Some immune elicitor proteins signal through structurally similar but phylogenetically diverse receptors in plants. The leucine-rich repeat receptor-like protein 30 (LRR-RLP30) in *Arabidopsis thaliana* is essential for defense against the necrotrophic fungus *Sclerotinia sclerotiorum* [34]. RLP30 recognizes a secreted, small cysteine-rich protein (SCP), a ligand broadly distributed across the fungal kingdom and also conserved in oomycetes such as *Phytophthora infestans* [35]. Remarkably, this SCP is also detected in the Solanaceae species *Nicotiana benthamiana* via a distinct receptor, LRR-RLP RE02, and in Brassica species by an yet unidentified receptor [35–37]. Different parts of SCP or the fully folded SCP are recognized via those diverse receptors, supporting independent evolution of receptor-ligand interaction. Notably, RLP30 also responds to a protein from bacterial Pseudomonads, demonstrating secondary ligand specificity for a molecule apparently sequence-unrelated to SCPs (Fig 1). When heterologously expressed in *Nicotiana tabacum*, RLP30 significantly reduces susceptibility to a broad spectrum of pathogens, including bacteria, fungi, and oomycetes.

Despite these shared functions, RLP30 and RE02 exhibit only ~24% sequence identity and lack a common ancestor RLP, suggesting that different plant families have evolved the ability to perceive the same elicitor protein through distinct, convergently evolved LRR-RLPs, as supported by phylogenetic analysis. The cross-kingdom conservation of SCPs as pathogen-associated molecular patterns (PAMPs), together with cross-family convergence of SCP receptor specificity, underscores the selective advantage of SCP sensitivity. Ultimately, RLP30 stands out for its capacity to detect a diverse array of ligands from bacteria, fungi, and oomycetes.

Different plant lineages independently evolved structurally distinct receptors to recognize the same or very similar elicitor molecules, illustrating evolutionary convergence in immune perception.

## CEP signaling—Complex receptor and ligand networks coordinating growth and immunity

Plant secreted immunogenic peptides are often organized in families that signal via a family of closely related receptors. C-terminally encoded peptides (CEPs) are widely conserved in gymnosperms and angiosperms, and play critical roles in root growth regulation and nutrient signaling [38–40]. Sequence diversification within their mature peptide domains classifies CEPs as canonical group I, non-canonical group I, and group II [41]. Canonical group I CEPs bind to CEP receptor 1 (CEPR1) and CEPR2, with CEPR1 being primarily genetically required for their regulation of systemic nitrogen-demand signaling and root development [39,40,42–44]. Recent studies show that both canonical and non-canonical group I CEPs can elicit PAMP-triggered immunity (PTI)-like responses, contributing to leaf immunity against bacterial pathogens [45]. Moreover, CEPs coordinate a cross-talk between cell surface immunity and nitrogen status [45]. Strikingly, recognition of non-canonical group I CEP4 involves not only CEPR1 but also CEPR2 and the phylogenetically related receptor RLK7 (Fig 1), with CEP4-induced responses showing distinct receptor dependencies [12].

Group II CEPs, though less characterized, are specifically recognized by CEPR2 and regulate systemic acquired resistance and cell surface immunity [46,47], revealing unexpected complexity within CEP perception systems. RLK7 also acts as a receptor for CEP-related, PAMP-induced peptides (PIPs) in various species [48–51]. Notably, CEP4 interacts with both RLK7 and CEPR2, whereas PIP1 only binds to RLK7, highlighting the intricate interplay between sequence-related peptides and receptors (Fig 1) [45]. Future research will need to unravel how CEPs and PIPs achieve such receptor and output specificity.

Tissue-specific promoter activity of CEPR1, CEPR2, and RLK7 suggests possible cooperation between peptide–receptor modules across tissues to ensure robust immunity. Although CEPs are mobile between root and shoot, CEP-mediated immune responses in the leaf require local expression, indicating a combination of local and systemic modes of action [39,45]. Ultimately, dissecting the spatiotemporal dynamics and specificities of CEPs, PIPs, and their receptors will be key to understanding their sophisticated regulation of plant immunity and developmental processes.

CEPs exemplify how plants use overlapping receptor and ligand modules to coordinate developmental and immune responses based on peptide type and tissue context.

## CERK1—Context-dependent signaling via dynamic receptor complexes

N-acetylated glycans serve as microbial patterns that plants detect by heterocomplexes of lysin (LysM) motif cell surface receptors. Chitin elicitor receptor kinase 1 (CERK1) is a LysM-RLK central to plant-microbe interactions, initially characterized in Arabidopsis for its role in fungal defense [52,53]. CERK1 exhibits remarkable versatility, primarily acting as a co-receptor with limited direct ligand-binding affinity, except for certain β-glucans, while relying on high-affinity receptors for microbial perception (Fig 1).

Rapid recognition of pathogens and activation of immune responses are essential for plant survival. In rice, fungal chitin is detected by the LysM-RLP Chitin Elicitor-Binding Protein (OsCEBiP), which binds chitin oligomers (e.g., octamers, CO_8_) and recruits CERK1 to initiate defense [54–57], aided by LysM-RLPs OsLYP4/6 [58]. In Arabidopsis, the kinase-inactive LysM-RLK AtLYK5 binds CO_8_ with high affinity and activates CERK1-mediated immunity [59], while AtLYK4 stabilizes this complex [60,61]. Legume CERK1 homologs likewise act in chitin‐triggered immunity [62]. AtCERK1 also functions as a co-receptor for unbranched β-1,3-glucans from fungal and oomycete cell walls [63] and, in both Arabidopsis and rice, likely as a *bona fide* receptor for β-1,3-1,4-glucans found in fungi, oomycetes, and grasses [64,65]. Moreover, CERK1 is involved in bacterial defense: It mediates peptidoglycan-induced immunity in concert with the LysM-RLPs AtLYM1/3 in Arabidopsis [66] or OsLYP4/6 in rice [58,67], and participates in lipopolysaccharide-triggered defense in rice [68].

However, there are further glycans and N-actelyated glycan derivatives that act as signaling molecules in plant-microbe interactions. Root mutualistic endosymbioses, such as arbuscular mycorrhiza (AM) with nutrient-acquiring fungi and root nodule symbiosis with nitrogen-fixing rhizobia, depend on microbial lipo-chitooligosaccharides (LCOs), which are recognized by specific LysM-RLKs like Nod Factor Perception (NFP; [69]) in *Medicago *truncatula**, thereby activating symbiosis signaling [70–72]. Strikingly, CERK1 also supports such beneficial plant–microbe interactions, supporting proper AM fungal colonization in rice and *M. truncatula* (Fig 1) [73–78].

This raises a central question: how is signaling specificity achieved despite CERK1’s versatility? Specificity likely derives from multiple factors, including ligand structure and length, receptor-complex composition, microdomain localization, dynamic gene expression, and post-translational modifications. In rice, for example, CO_8_ favors OsCEBiP-CERK1 immune signaling, while shorter CO_4_ oligomers promote OsMYR1-CERK1 symbiotic signaling [79]. In *M. truncatula*, simultaneous perception of LCOs involving MtNFP and of chitin/peptidoglycans involving CERK1 shifts transcriptional responses towards symbiosis [75]. Finally, signaling specificity also resides within the receptor itself; exchanging specific amino acid stretches of the *Lotus japonicus* CERK1 homolog CERK6 [62] with those of the LCO receptor Nod Factor Receptor 1 [80] confers LCO-binding capacity to CERK6 [81] and expression of the chimeric CERK6-NFR1 receptors restored symbiosis signaling in *nfr1* mutant plants [81,82].

Thus, CERK1 exemplifies how plants finely tune shared receptor networks to distinguish between friends and foes.

## Conclusion

Together, these examples underscore the remarkable diversity in the ligand recognition spectrum of plant cell surface receptors, which ranges from highly specific recognition of individual patterns to broad detection of endogenous and microbe-derived signals. Beyond ligand binding, the cellular context of receptors, including physico-chemical factors (e.g., pH), the presence of regulatory proteins such as accessory cell surface receptors, and membrane environment, further shape signaling outcomes. To fully understand this complexity, it is crucial to investigate the natural variation in plant cell surface receptor repertoires and to reconstruct the evolutionary trajectories that have shaped them. Equally important is elucidating the molecular and structural principles underlying ligand-binding specificity, and how individual receptors integrate multiple cues to coordinate diverse signaling pathways. Advancing this knowledge will provide a foundation for engineering crops with tailored immune responses and improved pathogen resistance.
